# Association between intraoperative blood salvage and coagulation disorder after cardiopulmonary bypass

**DOI:** 10.1186/s40981-024-00689-1

**Published:** 2024-01-25

**Authors:** Masahiro Morinaga, Kenji Yoshitani, Soshiro Ogata, Satsuki Fukushima, Hitoshi Matsuda

**Affiliations:** 1 Department of Anesthesiology, National Cerebral and Cardiovascular Center, Osaka, Japan; 2 Department of Preventive Medicine and Epidemiology, National Cerebral and Cardiovascular Center, Osaka, Japan; 3 Department of Cardiovascular Surgery, National Cerebral and Cardiovascular Center, Osaka, Japan

**Keywords:** Cardiac surgery, Cell salvage, Thromboelastgraphy, Coagulation disorder

## Abstract

**Background:**

This study investigated whether intraoperative blood salvage was associated with coagulation disorder diagnosed by conventional coagulation tests and thromboelastography (TEG) after cardiopulmonary bypass (CPB).

**Study design and methods:**

This was a prospective, observational study. Ninety-two patients who underwent cardiovascular surgery with CPB were enrolled. We evaluated coagulation function in patients with or without cell salvage blood transfusion at the following time points: before CPB, just after protamine administration, and 1 h after protamine administration. We evaluated platelet count, fibrinogen concentration, and TEG parameters. Patients were considered to have coagulation disorder if one or more of the following criteria were present: (1) residual heparin, (2) low platelet count, (3) low fibrinogen level, (4) low clotting factor level, and (5) hyperfibrinolysis.

**Results:**

Fifty-three of 92 patients (57.6%) received intraoperative cell salvage. Coagulation disorder was observed in 56 of 92 patients (60.9%) after CPB. There was no significant difference between patients with or without intraoperative blood salvage in terms of the incidence of coagulation disorder (*p* = 0.542) or the total volume of blood from the drain after CPB (*p* = 0.437). Intraoperative blood salvage was not associated with coagulation disorder diagnosed by either TEG or conventional coagulation tests (odds ratio 1.329, 95% confidence interval: 0.549–3.213, *p* = 0.547). There were no significant interactions between patients with or without intraoperative blood salvage regarding coagulation parameters derived from TEG.

**Conclusions:**

The incidence of coagulation disorder and the total blood volume from the drain after CPB did not differ significantly between patients with or without intraoperative blood salvage.

## Introduction

Blood products are in short supply worldwide [1], and therefore it is crucial to reduce their use. Cardiac surgery is associated with a significant need for blood transfusions due to cardiopulmonary bypass (CPB), which results in hemodilution and significant blood loss. In general, with a liberal threshold in cardiac surgery, three units of red blood cells are transfused per surgery [2], and it would be optimal if this number were reduced. Minimizing allogeneic blood transfusions is essential to prevent complications such as infection, immunomodulation, and transfusion-related acute lung injury [3]. One way to reduce blood transfusions is to use intraoperative blood salvage [4].

In cardiac surgery, blood in the CPB circuit remains after weaning from CPB, and blood is also salvaged from the operating field. Autologous salvaged blood can be transfused after it has been washed and concentrated. It has been reported that autologous salvaged blood transfusion reduces the use of allogeneic red blood cells and there is no significant difference between the two types of transfusion regarding the incidence of death or cardiovascular events [5]. On the other hand, there are reports that autologous salvaged blood transfusion leads to coagulation disorders [6–8]. Previous studies that reported the development of coagulation disorder related to cell salvage focused on high-risk patients [8]. By contrast, the present study focused on patients undergoing elective cardiac surgery with CPB. We hypothesized that the coagulation function of autologous salvaged blood would be similar to that of allogeneic blood in elective cardiac surgery.

We used viscoelastic monitoring with thromboelastography (TEG) and conventional coagulation tests to evaluate coagulation function after CPB [9], because a correlation between thromboelastography measurements and conventional laboratory tests has been reported in patients with poor coagulation function [10]. Furthermore, thromboelastography evaluates multiple aspects of clotting, including the rate of clot formation and clot strength. This study compared coagulation function and postoperative bleeding between patients who received intraoperative cell salvage and those who did not.

## Materials and methods

This prospective, observational study collected data from June 1, 2020, to September 30, 2021. Patients who underwent cardiac surgery with CPB were enrolled. Patients with congenital heart disease or aged < 15 years were excluded. Those with a planned surgery time > 8 h were also excluded because long CPB leads to coagulation disorder. After approval of our institutional review board (R20013-3), written informed consent was obtained. All patients underwent cardiac surgery with CPB using a cell salvage device (Cell Savor Elite+, Heamonetics, Boston, MA, USA, or CATSmart, Fresenius Kabi, Bad Homburg vor der Höhe, Germany), but some patients were not transfused with cell salvaged blood on the surgeon’s instructions. The patients were divided into two groups, specifically those who did or did not receive intraoperative blood salvage (only to produce autologous blood). Surgeons decided whether or not to use cell-salvaged blood based on factors such as CPB time, deep hypothermia, the administration of allogenic transfusion during CPB, and hemostatic state. In the cell salvage group, all blood resulting from bleeding before and after CPB was collected by cell salvaging; blood from bleeding during CPB was collected during cardiotomy and processed by cell salvaging after CPB. In patients without cell salvage, allogeneic transfusion was performed without blood processed by cell salvaging. Before CPB, we administered 300 units/kg of heparin and then checked whether the activated coagulation time (ACT) was more than 300 s. If the ACT was less than 300 s, 2000 units of heparin were added until the ACT surpassed 300 s., Protamine (3 mg/kg) was administered after CPB, and if the ACT was more than 150 s, an additional 20 mg of protamine was administered and the ACT was checked again. The decision to administer tranexamic acid or not was left to the discretion of the anesthesiologist in charge. When tranexamic acid was administered, the loading dose was 20 mg/kg and the maintenance dose was 2 mg/kg/h during surgery.

**Fig. 1:**
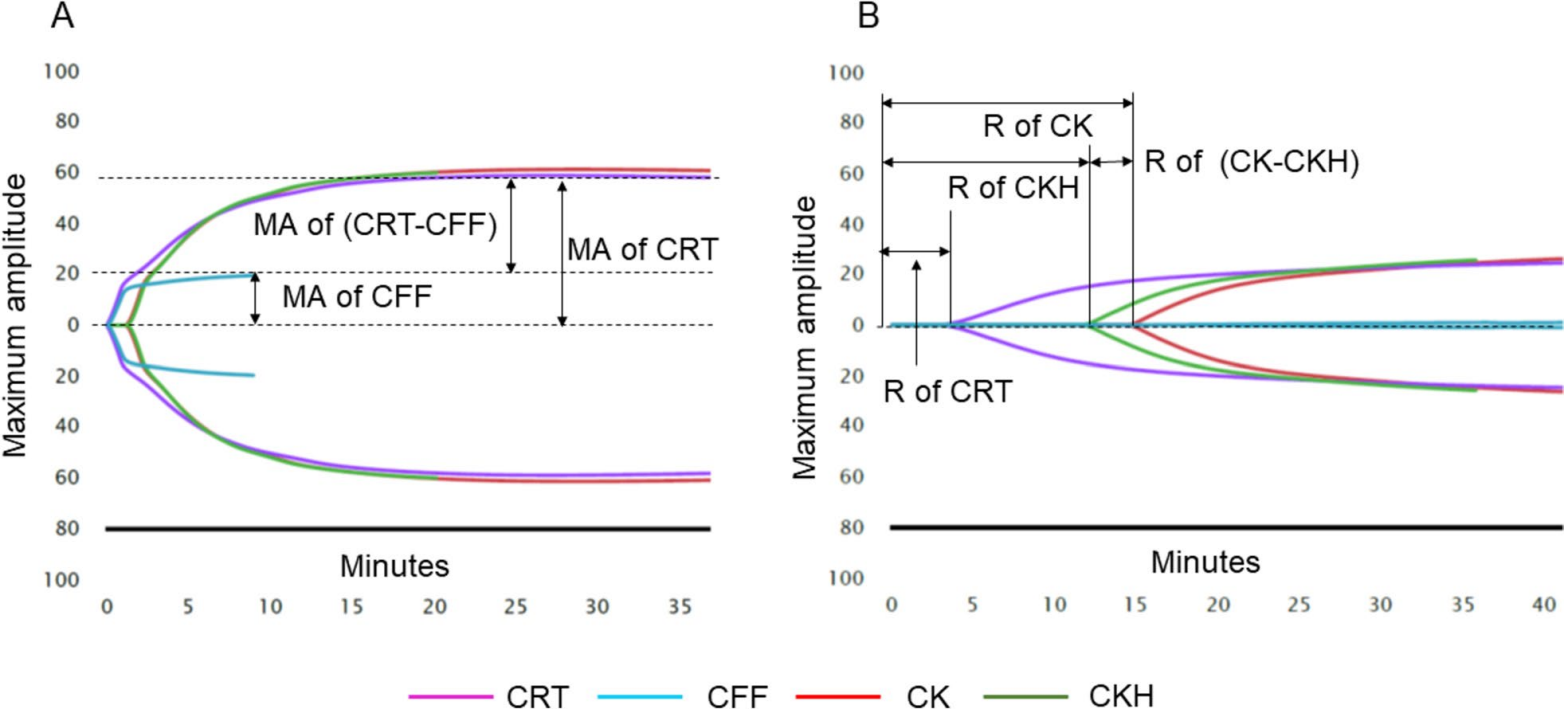
Typical results of thromboelastography. **a** Thromboelastography for normal coagulation function. **b** Thromboelastography for normal coagulation disorders. CFF: functional fibrinogen; CK: kaolin thromboelastography; CKH: heparinase kaolin thromboelastography; CRT: rapid thromboelastography; MA: maximum amplitude, which represents maximum clot strength. MA of (CRT−CFF) represents the coagulation function of platelets by subtracting the coagulation function of fibrinogen from that of whole blood. R: reaction time, which represents the time to initiation of clot formation. R of (CKH−CK) indicates the degree of residual heparin. Since CKH measures coagulation function by antagonizing heparin, subtracting CK represents the degree of residual heparin

Coagulation function was assessed at three-time points: before CPB, just after protamine administration, and 1 h after protamine administration. To comprehensively evaluate coagulation function, we employed both standard laboratory tests and thromboelastography, a blood viscoelasticity test. Standard laboratory tests included prothrombin time (PT), activated partial thromboplastin time (APTT), fibrinogen concentration, and platelet count. Four thromboelastography (TEG-6s) measurements were obtained: kaolin thromboelastography (CK), heparinase kaolin thromboelastography (CKH), rapid thromboelastography (CRT), and functional fibrinogen (CFF). Three parameters were evaluated for each of these: reaction time (R), which indicates the time from the start of the examination to initiation of clot formation; maximum amplitude (MA), which reflects maximum clot strength; and lysis rate-30 (LY30), which represents the rate of clot lysis for 30 min at MA (Fig. 1). To evaluate postoperative bleeding, we measured the volume of bleeding from the chest tube for 24 h.

## Primary endpoint

The primary endpoint of this study was the association between intraoperative blood salvage and coagulation disorder following CBP. Coagulation disorder was assessed using thromboelastography and standard laboratory tests [11–13] and was defined as a composite outcome consisting of residual heparin, low platelet count and function, low levels of fibrinogen and function, low clotting factor level, and hyperfibrinolysis. This multifaceted definition results in a comprehensive assessment and is a modified version of the composite outcome used in previous studies. Each element of the composite outcome is defined below.

1. Residual heparin

CK R > 2 × CKH R

Residual heparin can be detected using CKH, which measures coagulation function by antagonizing heparin. If heparin remains, CK R is expected to be greater than CKH R.

2. Low platelet count and function
  Platelet count < 100,000/μL or CRT MA−CFF MA < 35 mm
  Low platelet count and function can be diagnosed using either of the following criteria: a platelet count < 100,000/μL or a difference between the maximum amplitude (MA) of CRT (CRT MA) and the MA of the coagulation function of fibrinogen (CFF MA) < 35 mm. CRT MA−CFF MA indicates subtraction of the coagulation function of fibrinogen from that of whole blood and represents the coagulation function of platelets.
3. Low fibrinogen level and function
  Fibrinogen concentration < 1 g/L or CFF MA < 15 mm
  Low fibrinogen level and function can be diagnosed using either of the following criteria: a fibrinogen concentration < 1 g/L or a CFF MA < 15 mm.
4. Low clotting factor level
  CK R > 10 min
  A low clotting factor level can be diagnosed using CK, with a CK R > 10 min. CK utilizes kaolin to initiate coagulation, enabling a comprehensive assessment of coagulation function and verifying fundamental coagulation properties.
5. Hyperfibrinolysis
  CRT LY30 > 7.5%

Hyperfibrinolysis can be diagnosed using a CRT LY30 > 7.5%.

If one or more of the five criteria were met, the patient was considered to have coagulation disorder.

### Secondary endpoints

We assessed several secondary endpoints. First, we determined whether the volume of allogeneic transfusion products and postoperative bleeding from the chest tube differed between patients with or without cell-savage transfusion. Second, we compared the allogeneic and intraoperative blood salvage groups in terms of the total number of autologous red blood cells and transfused platelets and the total amount of fresh frozen plasma and blood from the chest tube. Third, we investigated potential interactions between the two groups regarding the variables used to diagnose coagulation disorder, including hemoglobin concentration, PT, APTT, platelet count, CRT MA, CFF MA, CRT MA–CFF MA, CRT LY30, CK R, CKH R, and CK R−CKH R.

### Statistics

This was a preliminary, prospective study. Therefore, we did not perform sample size calculations. We examined whether intraoperative blood salvage was associated with coagulation disorder diagnosed based on residual heparin, low platelet count, low levels of fibrinogen or clotting factors, and hyperfibrinolysis. We adjusted for possible confounders such as age, sex, CPB time, redo surgery, aortic surgery, preoperative anticoagulation, antiplatelet therapy, and surgeons who performed the procedure (10 main surgeons vs. others) in a multivariable logistic regression analysis of the primary endpoint. To prevent overfitting of the logistic regression, we tested the association between intraoperative blood salvage and coagulation disorder, adjusting only for age and gender. In the analyses of the secondary endpoints, we used a mixed-effects model with patients as the random intercept to identify interactions between variables used to diagnose coagulation disorder. For the analysis of continuous variables, we used the Wilcoxon rank-sum test. We compared categorical variables using the chi-square test. We considered *p* < 0.05 as statistically significant. We performed all statistical analyses with Stata 17 SE.

## Results

Patients were enrolled in this study from June 2020 to September 2021. Those who did not receive a homologous transfusion at the end of CPB were excluded. A total of 381 patients were deemed eligible for this study; of these, 85 were excluded because they did not provide informed consent, and 204 were excluded because they had a planned surgery time > 8 h. Finally, 92 patients were included in this study, of whom 53 underwent intraoperative blood salvage and 39 underwent autologous transfusion. Coagulation disorder was observed in 56 of 92 patients (60.9%) after CPB.

Table 1 presents the patient characteristics of the two groups, receiving intraoperative blood salvage and allogeneic transfusion. There were no significant differences between the two groups.

**Table 1:** Patient characteristics of the two groups

	Intraoperative blood salvage (*n* = 53)	Allogeneic transfusion (*n* = 39)	*p* value
Age (years)	73 (67, 78)	72 (60, 77)	0.34
Male sex	35 (66%)	24 (62%)	0.66
Height (cm)	164 (157, 170)	165 (152, 169)	0.96
Weight (kg)	62 (55, 69)	61 (52, 68)	0.92
Hypertension	35 (66%)	23 (59%)	0.49
Diabetes mellitus	5 (9%)	3 (8%)	0.77
Anticoagulation	12 (23%)	15 (38%)	0.10
Antiplatelet	11 (21%)	7 (18%)	0.74
Redo surgery	10 (19%)	10 (26%)	0.44
Aortic surgery	14 (26%)	14 (36%)	0.33
Valvular surgery	37 (70%)	24 (62%)	0.41

Data are presented as medians (interquartile range) for age, height, and weight

Table 2 summarizes the coagulation status of the two groups. Coagulation disorder occurred in 56 (34 and 22 with/without intraoperative blood salvage, respectively) of 92 patients overall. The two groups did not differ significantly regarding any of the five coagulation disorder components. The incidence of re-exploration within 24 h was not significantly different between the two groups.

**Table 2:** Coagulation status in the intraoperative blood salvage and allogeneic transfusion groups

	Intraoperative blood salvage (*n* = 53)	Allogeneic transfusion (*n* = 39)	*p* value
Residual heparin (*n*)	1	2	0.387
Low platelet count (*n*)	22	15	0.768
Low fibrinogen (*n*)	1	0	0.388
Low clotting factor (*n*)	16	12	0.952
Hyperfibrinolysis (*n*)	0	0	n.s.
Coagulopathy (*n*)	34	22	0.452
re-exploration (*n*)	3	0	0.131

Residual heparin: reaction time (*R*) for kaolin TEG (CK) less than half of R for kaolin TEG with heparinase (CKH); low platelet: platelet count < 100,000/μL or rapid TEG (CRT) maximum amplitude (MA)−TEG functional fibrinogen (CFF) MA < 35 mm; low fibrinogen: R for CK > 10 min

Table 3 shows the total volume of allogeneic blood transfusion and blood from the chest tube for 24 h and during the ICU stay. Neither parameter differed significantly between groups.

**Table 3:** Allogeneic transfusion and bleeding

	Intraoperative blood salvage (*n* = 53)	Allogeneic transfusion (*n* = 39)	*p* value
RBC (mL)	560 (0–840)	560 (560–840)	0.066
FFP (mL)	480 (0–720)	480 (480–960)	0.066
Platelets (mL)	400 (0–500)	400 (0–400)	0.419
Blood from the drain for 24 h (mL) in the ICU	825 (625–1195)	735 (495–960)	0.200
Blood from the drain during the ICU stay (mL)	1288 (765–3563)	1103 (665–2561)	0.437

Data are presented as medians (interquartile range)

*FFP* fresh frozen plasma, *RBC* red blood cell

**Fig. 2:**
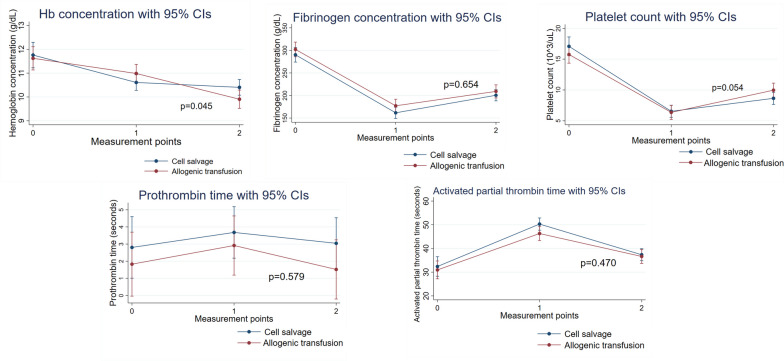
Time course of changes in hemoglobin concentration, platelet count, and conventional hemostatic variables in the cell salvage and allogeneic transfusion groups. Measurement points: 0, before cardiopulmonary bypass; 1, just after the administration of protamine; 2, 1 h after the administration of protamine. CI: confidence interval

**Fig. 3:**
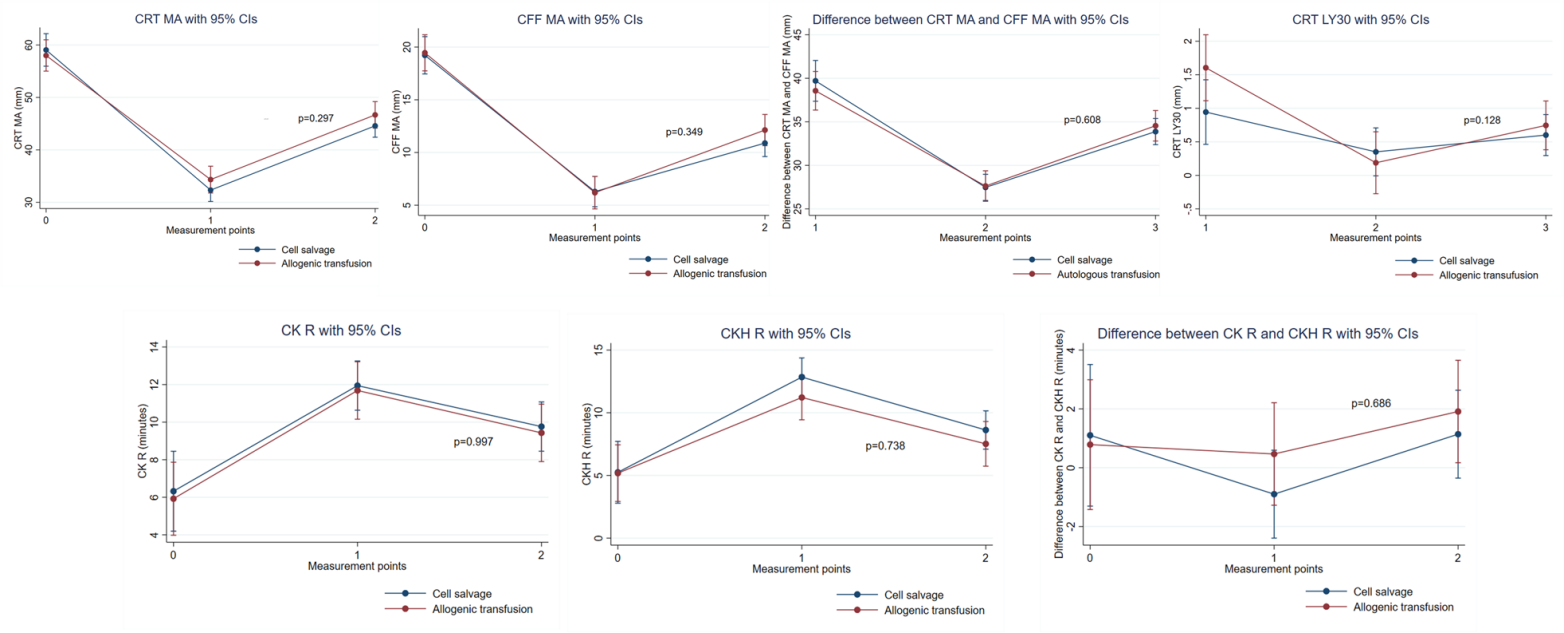
Time course of changes in thromboelastography parameters in the cell salvage and allogeneic transfusion groups. Measurement points: 0, before cardiopulmonary bypass; 1, just after the administration of protamine; 2, 1 h after the administration of protamine. CFF: TEG functional fibrinogen; CI: confidence interval; CK: kaolin TEG; CKH: heparinase kaolin TEG; CRT: rapid TEG; LY30: lysis rate for 30 min; MA: maximum amplitude; R: reaction time

Figure 2 depicts the interactions between intraoperative blood salvage and allogeneic transfusion groups in terms of hemoglobin concentration, platelet count, fibrinogen concentration, PT, and APTT. There was a significant interaction only for hemoglobin concentration (*p* = 0.045), suggesting that it differed significantly by intraoperative blood salvage status. Figure 3 shows that there were no interactions between the two groups in any of the following: CRT MA; CFF MA; CRT MA - CFF MA; LY30 of CRT; CK R; CKH R; CK R-CKH R. This indicates that these parameters did not differ significantly by intraoperative blood salvage status.

Table 4 demonstrates that intraoperative blood salvage was not associated with coagulation disorder (odds ratio, 1.343; 95% confidence interval, 0.540–3.341; *p* = 0.525). To test whether overfitting occurred in the logistic regression analysis in Table 4, we examined the association of intraoperative blood salvage and coagulation disorder adjusted by only age and sex was similar (odds ratio, 1.36; 95% confidence interval, 0.584–3.186; *p* = 0.474) with that in Table 4.

**Table 4:** Characteristics associated with coagulation disorder after intraoperative blood salvage

	Odds ratio	*p* value	95% CI
Age	1.008	0.683	0.969–1.049
Sex	1.168	0.717	0.444–3.071
Anticoagulation therapy	0.766	0.546	0.268–2.191
Antiplatelet therapy	1.158	0.826	0.346–3.869
CPB time	0.999	0.796	0.990–0.007
Aortic surgery	1.113	0.860	0.338–3.668
Redo	1.141	0.824	0.357–3.646
Cell salvage	1.329	0.547	0.549–3.213

*CI* confidence interval, *CPB* cardiopulmonary bypass

## Discussion

Intraoperative blood salvage was not found to be associated with coagulation disorder diagnosed by thromboelastography and conventional tests of coagulation function. There were no significant differences in the incidences of the five components of the composite outcome we used to define coagulation. The total blood volume from the chest tube was similar between patients with or without cell salvage after CPB. There was a significant interaction between the two groups in hemoglobin concentration but not in the other conventional coagulation parameters.

The possibility of coagulation disorder occurring because of intraoperative blood salvage has previously been discussed. Recently, Adam and colleagues reported a significant reduction in coagulation factors during the cell salvage-washing process [14]. However, they did not evaluate the systemic coagulation status after transfusing cell-salvaged blood. Palla and colleagues reported that to achieve successful hemostasis, trough levels of coagulation factors must be at least 10–30% of their normal level [15]. Therefore, lower concentrations of coagulation factors do not always lead to coagulation disorder.

Rollins and colleagues reported a case of coagulation disorder associated with massive intraoperative blood salvage following aortic surgery [16]. In that case, the cell-salvaged blood that was transfused was obtained from the mediastinal drains connected to the cell-saver system. The authors suggested that an excessive dose of heparin was administered before processing, resulting in a high APTT. Residual heparin from the cell saver should be considered a reason for coagulation disorder.

Niranjan and colleagues demonstrated no significant differences in coagulation markers, PT, APTT, and total blood volume from the chest tube between patients with or without intraoperative blood salvage in a randomized controlled trial [17]. These results are consistent with those of our study, suggesting that there might be no differences in coagulation function resulting from cell salvage or autologous transfusion.

On the other hand, Xie and colleagues demonstrated that the incidences of excessive postoperative bleeding, residual heparin, and total impairment of blood coagulation were significantly higher in patients with intraoperative blood salvage than in those without [8]. Their study enrolled patients at high risk of bleeding who had liver dysfunction, hemoglobin levels < 13.0 g/dL, platelet counts < 50 × 10^9^/L, and aspirin intake within 3 days or clopidogrel within 7 days before surgery, as these factors were considered by the authors to potentially cause coagulation disorder. By contrast, our study included patients who underwent CPB, which led to different results; there were no significant differences in coagulation disorder between patients with or without intraoperative blood salvage, possibly because the underlying risk in all patients was lower than in those recruited by Xie and colleagues.

One strength of this study is the use of thromboelastography to evaluate coagulation function after CPB. Conventional coagulation tests have limitations in assessing dynamic coagulation function in cardiac surgery. Thromboelastography evaluates all components of the coagulation cascade in whole blood and can also assess total clot strength and the contribution of platelets and fibrinolysis [9]. We believe that our results reflect clinically relevant coagulation function.

This study has several limitations. First, the number of patients was small because this was a preliminary study. We believe that this study will be significant in terms of facilitating sample size calculations in future RCTs and providing new insight into adverse events of intraoperative blood salvage and associated confounding factors. The results provide a rationale for conducting an RCT to evaluate whether intraoperative blood salvage leads to coagulation disorder. Second, this was a prospective, observational study. Although we adjusted for known possible confounders, there might be residual bias due to unknown confounders.

In conclusion, no significant associations existed between intraoperative blood salvage and coagulation disorder after CPB.

## Data Availability

The datasets used and analyzed during the current study are available from the corresponding author on reasonable request.

## References

[1] Roberts N, James S, Delaney M, Fitzmaurice C (2019). The global need and availability of blood products: a modelling study. Lancet Haematol.

[2] Mazer CD, Whitlock RP, Fergusson DA, Hall J, Belley-Cote E, Connolly K, Khanykin B, Gregory AJ, De Médicis É, McGuinness S, Royse A, Carrier FM, Young PJ, Villar JC, Grocott HP, Seeberger MD, Fremes S, Lellouche F, Syed S, Byrne K, Bagshaw SM, Hwang NC, Mehta C, Painter TW, Royse C, Verma S, Hare GMT, Cohen A, Thorpe KE, Jüni P, Shehata N (2017). Restrictive or liberal red-cell transfusion for cardiac surgery. N Engl J Med.

[3] Kilic A, Whitman GJ (2014). Blood transfusions in cardiac surgery: indications, risks, and conservation strategies. Ann Thorac Surg.

[4] Vonk AB, Meesters MI, Garnier RP, Romijn JW, van Barneveld LJ, Heymans MW, Jansen EK, Boer C (2013). Intraoperative cell salvage is associated with reduced postoperative blood loss and transfusion requirements in cardiac surgery: a cohort study. Transfusion.

[5] Wang G, Bainbridge D, Martin J, Cheng D (2009). The efficacy of an intraoperative cell saver during cardiac surgery: a meta-analysis of randomized trials. Anesth Analg.

[6] Rollins KE, Trim NL, Luddington RJ, Colah S, Klein A, Besser MW, Nair SK (2012). Coagulopathy associated with massive cell salvage transfusion following aortic surgery. Perfusion.

[7] Scrascia G, Rotunno C, Nanna D, Rociola R, Guida P, Rubino G, de Luca Tupputi Schinosa L, Paparella D. (2012). Pump blood processing, salvage and re-transfusion improves hemoglobin levels after coronary artery bypass grafting, but affects coagulative and fibrinolytic systems. Perfusion.

[8] Xie Y, Shen S, Zhang J, Wang W, Zheng J (2015). The efficacy, safety and cost-effectiveness of intra-operative cell salvage in high-bleeding-risk cardiac surgery with cardiopulmonary bypass: a prospective randomized and controlled trial. Int J Med Sci.

[9] Haas T, Fries D, Tanaka KA, Asmis L, Curry NS, Schochl H (2015). Usefulness of standard plasma coagulation tests in the management of perioperative coagulopathic bleeding: is there any evidence?. Br J Anaesth.

[10] Lloyd-Donald P, Vasudevan A, Angus P, Gow P, Martensson J, Glassford N, Eastwood GM, Hart GK, Jones D, Weinberg L, Bellomo R (2020). Comparison of thromboelastography and conventional coagulation tests in patients with severe liver disease. Clin Appl Thromb Hemost.

[11] Diprose P, Herbertson MJ, O’Shaughnessy D, Deakin CD, Gill RS (2005). Reducing allogeneic transfusion in cardiac surgery: a randomized double-blind placebo-controlled trial of antifibrinolytic therapies used in addition to intra-operative cell salvage. Br J Anaesth.

[12] Shen S, Zhang J, Wang W, Zheng J, Xie Y. Impact of intra-operative cell salvage on blood coagulation in high-bleeding-risk patients undergoing cardiac surgery with cardiopulmonary bypass: a prospective randomized and controlled trial. J Transl Med. 2016;14 10.1186/s12967-016-0986-6 PMC4966771 27473326

[13] Shore-Lesserson L, Manspeizer HE, DePerio M, Francis S, Vela-Cantos F, Ergin MA (1999). Thromboelastography-guided transfusion algorithm reduces transfusions in complex cardiac surgery. Anesth Analg.

[14] Adam EH, Funke M, Zacharowski K, Meybohm P, Keller H, Weber CF (2020). Impact of intraoperative cell salvage on blood coagulation factor concentrations in patients undergoing cardiac surgery. Anesth Analg.

[15] Palla R, Peyvandi F, Shapiro AD (2015). Rare bleeding disorders: diagnosis and treatment. Blood.

[16] Campbell J, Holland C, Richens D, Skinner H (2012). Impact of cell salvage during cardiac surgery on the thrombelastomeric coagulation profile: a pilot study. Perfusion.

[17] Niranjan G, Asimakopoulos G, Karagounis A, Cockerill G, Thompson M, Chandrasekaran V (2006). Effects of cell saver autologous blood transfusion on blood loss and homologous blood transfusion requirements in patients undergoing cardiac surgery on- versus off-cardiopulmonary bypass: a randomised trial. Eur J Cardiothorac Surg.

